# Cognitive assessment instruments in Parkinson's disease patients undergoing deep brain stimulation

**DOI:** 10.1590/S1980-57642012DN06010002

**Published:** 2012

**Authors:** Aline Juliane Romann, Silvia Dornelles, Nicole de Liz Maineri, Carlos Roberto de Mello Rieder, Maira Rozenfeld Olchik

**Affiliations:** 1 Mestranda em Medicina, Ciências Médicas, Universidade Federal do Rio Grande do Sul, Porto Alegre RS, Brazil (UFRGS). Fonoaudióloga Clínica.; 2 Doutora em Ciências da Criança e do Adolescente, UFRGS. Professora Adjunto da UFRGS, Departamento de Psicologia do Desenvolvimento e da Personalidade.; 3 Mestre em Medicina e Ciências da Saúde, Pontifícia Universidade Católica do Rio Grande do Sul, Porto Alegre RS, Brazil (PUCRS). Neuropsicóloga do Laboratório de Estudos Cognitivos, MemoLab (Hospital Moinhos de Vento).; 4 Doutor em Clinical Neuroscience (University of Birmingham). Professor Adjunto de Neurologia da Universidade Federal de Ciências da Saúde de Porto Alegre (UFCSPA) e do Programa de Pós Graduação em Medicina, Ciências Médicas, UFRGS.; 5 Doutora em Educação, UFRGS. Professora Adjunto do Curso de Fonoaudiologia da UFRGS, Departamento de Cirurgia e Ortopedia.

**Keywords:** Parkinson's disease, cognition, deep brain stimulation

## Abstract

Deep Brain Stimulation (DBS) is a widely used surgical technique in individuals with Parkinson's disease (PD) that can lead to significant reductions in motor symptoms.

**Objectives:**

To determine, from publications, the most commonly used instruments for cognitive evaluation of individuals with PD undergoing DBS.

**Methods:**

A systematic review of the databases: PubMed, Medline, EBECS, Scielo and LILACS was conducted, using the descriptors "Deep Brain Stimulation", "Verbal Fluency", "Parkinson Disease", "Executive Function", "Cognition" and "Cognitive Assessment" in combination.

**Results:**

The Verbal Fluency test was found to be the most used instrument for this investigation in the studies, followed by the Boston Naming Test. References to the Stroop Test, Trail Making Test, and Rey's Auditory Verbal Learning Test were also found.

**Conclusions:**

The validation of instruments for this population is needed as is the use of batteries offering greater specificity and sensitivity for the detection of cognitive impairment.

## Abstract (pt)

A Estimulação Cerebral Profunda (ECP) tem sido uma técnica cirúrgica bastante utilizada devido à redução significativa dos sintomas motores.

**Objetivos:**

Verificar, a partir das publicações, quais os instrumentos mais utilizados para avaliação cognitiva em pacientes com DP submetidos à ECP.

**Métodos:**

Foi realizado uma revisão sistemática nas bases de dados PubMed, Medline, EBECS, Scielo e LILACS utilizando os descritores "Deep Brain Stimulation", "Verbal Fluency", "Parkinson Disease", "Executive Function", "Cognition" e "Cognitive Assessment" de forma combinada.

**Resultados:**

O teste de Fluência Verbal o instrumento mais utilizado para esta investigação nos estudos encontrados, seguido pelo Teste de Nomeação de Boston. Foram também encontradas referências aos testes Teste de Stroop, Teste das Trilhas, Teste de Aprendizado Auditivo Verbal de Rey.

**Conclusões:**

A validação de instrumentos para esta população se faz necessária bem como a utilização de baterias com mais especificidade e sensibilidade para detecção das alterações cognitivas nesta população.

## INTRODUCTION

Tremor, bradykinesia and rigidity are the main sources of discomfort reported by Parkinson's disease patients. In addition to the motor symptoms that affect activities of daily living (ADL) and the quality of communications and eating, PD has other symptoms. Cognitive changes occur, in most cases, in the more advanced stages of the disease, preceded by psychiatric signs, such as hallucinations and psychosis.^1^ Other symptoms such as depression, may be present from the early stages of PD.^2^

Many treatment options have been developed since the discovery of this disease, such as new medications, technology and surgical techniques, as well as rehabilitation. However, drugs do not mitigate or delay disease symptoms, and a cure for PD remains an elusive challenge among researchers.

Deep Brain Stimulation (DBS) is a stereotactic technique in which two leads fitted with four electrodes are implanted in the region of the basal ganglia. The treatment is indicated for patients with PD undergoing treatment with drugs and who present with further complications, such as dyskinesia, motor fluctuations and refractory tremor.^3^ The motor gains acquired as a result of the neuronal inhibition provided by the neurostimulator are significant.^4^ However, DBS may have a negative impact on both communication skills^5^ and cognitive symptoms.^6,7^

In 2007, the Movement Disorder Society (MDS) proposed criteria and instruments for diagnosing and classifying dementia in Parkinson Disease patients (PD-D). As the screening tool for diagnosing PD-D, the MSD proposed the Mini Mental State Examination (MMSE); the Months Reversed, Lexical Fluency (LV) and Clock Drawing (CD) tests. To provide a more detailed series of assessments allowing characterization of the components of PD-D, the MSD recommended the following tests: the Mattis Dementia Rating Scale (MDRS), Digit Span (DS), Spatial Span from the CANTAB, Digit Ordering (DO), Similarities of the WAIS-R, Wisconsin Card Sorting Test (WCST), Verbal Fluency (VF), Trail Making Test (TMT), Stroop Test (ST), Odd Man Out Test (OMO), Prehension Behavior (PB), Apathy Scale (AS), the Neuropsychiatric Inventory (NPI), Montgomery and Asberg Depression Rating Scale (MADRS), Hamilton Depression Rating Scale (HDRS), Beck Depression Inventory (BDI), Geriatric Depression Scale (GDS), Parkinson Psychosis Questionnaire (PPQ), Rey Auditory Verbal Learning Test (RAVL), Free and Cued Recall Test (FC), Boston Naming test (BNT), Benton Line Orientation Test (BLO) and Benton Face Recognition Test (BFO) or the Fragmented Letters of the VOSP.^8^

The American Academy of Neurology (AAN) listed instruments for the cognitive and neuropsychiatric examination of individuals with PD. To investigate psychiatric symptoms, the AAN suggested the use of the BDI, the HDRS, the Hamilton Anxiety Rating Scale (HARS), the Brief Psychiatric Rating Scale (BPRS), the Schedule for the Assessment of Positive Symptoms (SAPS), the AS and NPI; whereas for cognitive investigations, the AAN suggested the Cambridge Cognitive Examination (CAMCog), the Alzheimer's Disease Assessment Scale-cognitive (ADAS COG), the Addenbrooke's Cognitive Examination - Revised (ACE-R), the Clinician Global Impression of Change (CGIC), the Montreal Cognitive Assessment (MoCA), the MDRS, the Parkinson Neuropsychometric Dementia Assessment (PANDA), the Parkinson's Disease-Cognitive Rating Scale (PD-CRS), the Scales for Outcomes of Parkinson's Disease - Cognition (SCOPA-COG), the Short Portable Mental Status Questionnaire (SPMSQ) and the MMSE.^9^ However, patients that undergo DBS implantation can have a wide variety of cognitive symptoms, and the best instruments for evaluation are not well established.

This study identified which instruments are used for the cognitive evaluation of patients with PD undergoing DBS.

## METHODS

A systematic review of the literature was conducted including all publications available in PUBMED, MEDLINE, LILACS, EBECS and SCIELO. In order to conduct a broad-based literature review, the search included studies published since 1997, representing the date of the first publication evaluating cognitive aspects of patients with PD in the databases reviewed. The search was conducted using the following key words: " *Parkinson disease* " and " *deep brain stimulation* " combined with " *verbal fluency ", " cognition ", " executive function* " or " *cognitive assessment* ".

The studies, reviewed independently by three examiners, were selected according to the following inclusion criteria:

- Published between 1997 and 2011;
- Original studies involving human beings;
- Studies whose objective was the cognitive assessment of patients with PD that received unilateral or bilateral DBS;
- At least one instrument of cognitive assessment;
- Positive or negative cognitive results.

Studies not meeting these criteria were excluded. To ensure that all examiners had the same criteria to evaluate abstracts, a data collection form (Appendix A) with the criteria described above was devised and filled out for each study. Each assessor assigned a grade from 0 to 10 to each study. Studies graded below 8 were excluded from this review.

## RESULTS

A total of 523 studies were found in the databases used of which 473 were excluded: 258 because they were the same study indexed in different databases, and 215 due to other exclusion criteria. Fifty studies were included in this review,^6,7,10-58^ and 90 instruments were found: 71 tests and 19 scales. Given the large number of instruments, only those used in more than 10 of the studies included in this review are described below. All inventories and scales found are listed in Tables 1 and 2. To facilitate comprehension, and because the same instrument might evaluate more than one cognitive aspect, instruments were classified according to the predominant cognitive function assessed: attention, perception, memory, language, dexterity, executive functions, cognitive screening tests, intelligence, and laterality in handedness. Similarly, scales were classified according to whether they investigated depression, anxiety, mood, apathy, psychiatric disorders and quality of life. This classification was based on the suggestions proposed by Lezak (2004).^59^

**Table 1:** Tests and Cognitive Screening Batteries used in the studies, classified according to the predominant cognitive function tested.

Cognitive function	Tests	Number of studies
**Attention**	Stroop Test	21
	Trail Making Test	20
	Corsi's Block Tapping Test	8
	Go-No-Go Task	3
	Symbol Digital Modalities Test	1
	N-Back Task	1
	Oral Trail Making Test	1
	Spinler Matrices Test	1
	Color Word Interference Test (D-KEFS)	1
	Visual Reaction Time (Vrt)	1
	Money's Standardised Road Map Test for Direction Sense (Mrmt)	1
	Test For Attentional Performance (Tap)	1
**Visual Perception**	Hooper Visual Organizational Test	2
	The Visual Object and Space Perception Battery (Vosp)	1
**Memory**	Rey's Auditory Verbal Learning Test	14
	Digit Span	11
	Wechsler Memory Scale	6
	Paired Associate Learning	4
	Benton Visual Retention Test	4
	Grober And Buschke Verbal Learning Test	3
	Benton Line Orientation Test	3
	Random Number Generation Task (Rngt)	2
	California Verbal Learning Test	2
	Hopkins Verbal Learning Test	2
	Logical Memory Task	2
	Rey Osterrieth Complex Figure Test	2
	Brief Visuospatial Memory Test	1
	Visual Conditional Learning Test	1
	Benton Judgment Of Line Orientation Test	1
	Rey Figure/Taylor Figure	1
	Memory Assessment Clinics Rating (Mac)	1
	Rivermead Behavioural Memory Test	1
	Rey-Kim Memory Battery	1
	Brief Visual Memory Test	1
	Conditional Associative Learning Test (CALT)	1
	Externally Ordered Working Memory Test	1
**Language**	Verbal Fluency	37
	Boston Naming Test	12
	Controlled Oral Word Association Test	6
	Bi-Syllabic words Repetition Test	6
	Boston Diagnostic Aphasia Examination	1
	Regensburg Word Fluency Test (RWT)	1
	Syntactic Comprehension Test and Morphological Test	1
	Agnosia Screening Task of Schnider	1
	ABBA	1
	North American Adult Reading Test	1
**Construct**	Clock Drawing	1
	Grooved Pegboard Test	1
	Purdue Pegboard Test	1
**Executive functions**	Wisconsin Card Sorting Test	17
	Raven's Progressive Matrices	10
	Modified Wisconsin Card Sorting Test	4
	Frontal Assessment Battery	3
	Tower of London	2
	Dex Questionnaire of the Behavioural Assessment of the Dysexecutive Syndrome	1
	Paced Auditory Serial Addition Task	1
	Paced Visual Serial Addition Test	1
	Frontal systems behavior scale	1
	Odd Man Out Test	1
	Vocabulary and Reasoning of The "Leistungsprufsystem" (LPS)	1
	Homophone Meaning Generation Test	1
**Cognitive Screening Batteries**	Mini Mental State Examination	18
	Mattis Dementia Rating Scale	15
	Dementia Rating Scale	7
	Dutch Adult Reading Test	1
	National Adult Reading Test	1
	Cerad Neuropsychological Battery	1
**Intelligence**	Wechsler Adult Intelligence Scale	7
	Groningen Intelligence Test	1
	Verbal Intelligence Quotient	1
**Laterality of Handedness**	Edinburgh Handedness Inventory	1

**Table 2:** Scales used in the studies

	Scales	Number of studies
**Depression**	Beck Depression Inventory	20
	Montgomery-Asberg Depression Rating Scale	6
	Hamilton Depression Rating Scale	2
	Geriatric Depression Scale	1
	Hospital Anxiety And Depression Scale	1
	Brief Symptom Inventory	1
**Anxiety**	State-Trait Anxiety Inventory	4
	Beck Anxiety Inventory	2
	Hamilton Anxiety Rating Scales	1
	Snaith-Hamilton pleasure Scale	1
	Maudsley Obsessional Compulsive Inventory	1
**Mood**	Visual Analogue Mood Scale	1
	Positive And Negative Affect Scale	1
	Profile Of Mood States	1
**Apathy**	Apathy Scale	1
	Apathy Evaluation Scale	1
**Psychiatric**	Neuropsychiatric Inventory	2
	Bech-Rafaelsen Mania Scale	1
**Quality of life**	Parkinson's Disease Questionnaire (PDQ-39)	3

### Cognitive assessment tests of PD patients undergoing DBS

*Attention tests -* The instrument most often used for attention was the Stroop Test (ST), found in 21 studies. This test, developed by John Ridley Stroop in 1935, is aimed at evaluating selective attention, inhibitory capacity and concentration. This test has some variations, but the full format has the following stages. Scores may be defined according to test performance time, number of errors or both, or according to the number of items read or named within a given timeframe.^59^

The ST was standardized by Tosi (2004)^59^ for use in Brazilian populations, and was tested for Brazilian students aged 12 to 14 years to obtain normative data for this population.^61^ However, this test has not been standardized for adult and elderly Brazilians, nor validated for individuals with PD.

Cognitive aspects, such as visual attention, processing speed, flexibility and planning, have been evaluated in 20 studies using the Trail Making Test (TMT), which originated from the Army Individual Test Battery (1944) and has two parts, A and B. The score is defined according to time taken, i.e. the test should be completed as fast as possible.^59^

The TMT was used for a Brazilian population by Hamdan and Hamdan (2009),^62^ who found that age and schooling affected individual scores, and that there was a significant increase in the time required to complete the TMT tasks according to the individual's age. Also, mean time taken decreases with greater schooling.

*Memory test -* The Digit Span (DS) test, part of the Wechsler Adult Intelligence Scale (WAIS), was used individually in 11 studies, and together with the WAIS in 7 studies. Scores are defined by the number of correct answers. Adults without deficits are expected to repeat at least 5 numbers in direct order and at least 3 in inverse order. Age tends to affect performance only for individuals >65 years old, for whom the normal score is 5 right answers.^59^

The DS test alone has not been standardized for Brazilian populations or individuals with PD. However, together with the WAIS, it was standardized for a Brazilian population by Nascimento et al. (1998)^63^ and validated for a population with PD by Randolph et al. (1993).^64^

A test used in 14 studies to evaluate immediate, short- and long-term memory was the Rey's Auditory Verbal Learning Test (RAVLT), developed by Rey in 1964. This test has several variations, but the one most often used consists of three parts. The RAVLT learning score is the sum of words remembered, with the maximum score again 15, and the closer the result to this number, the better the performance.^59^

In Brazil, Malloy-Diniz et al. (2007)^65^ developed a version of RAVLT with nouns frequently used in Portuguese, and applied it to groups of elderly individuals aged 60 to 89 years. The authors found that the Brazilian adaptation of the RAVLT was appropriate and evaluated the memory of Brazilian individuals of the same age and schooling. In 2009, Teruyaet al.^66^ conducted a pilot study to evaluate the performance of normal Brazilian adults using the RAVLT. The group found that overall test performance decreased as age increased, and that schooling was positively associated with scores. In 2010, Fichmanet al.^67^ published a study to validate the RAVLT. The authors found a strong association between episodic memory and sociodemographic variables. This finding is relevant in a country like Brazil, where educational levels vary substantially. However, further studies should be conducted to test the effect of age and schooling on RAVLT performance.

*Language tests -* The Verbal Fluency test, used in 37 studies, was the most frequent test employed to evaluate executive functions, language and semantic memory. The phonological VF (FAS) prompts the respondent to name words starting with the letters F, A and S, excluding proper names, numbers, the same word with different suffixes, or different conjugations of the same verb. A time of 1 minute is assigned for each letter.^59^ The test was normalized by Tombaugh et al. (1998)^68^ but no studies have been found on FAS validation for populations with PD.

The VF test has a variation, a semantic restriction, in which the individual has 1 minute to produce words limited to one semantic class (animals, fruit, foods, etc.). This instrument variation evaluates the capacity to search and retrieve data stored in long-term memory within a certain category and to demonstrate the capacities of organization, self-regulation and operational memory. This variation was validated for the Brazilian population by Brucki et. al (1997;2004).^69,70^

The Boston Naming Test (BNT) was used in 12 studies to evaluate language skills and semantic memory by naming figures. Developed by Kaplan, Goodglass and Weintraub in 1978, it consists of the presentation of 60 items drawn in black and white, graded according to difficulty parameters, which the individual has to name spontaneously in 20 seconds. This test was validated for a Brazilian population by Miotto et al. (2010).^71^ In this adapted version, the authors replaced 20 figures to take into consideration cultural familiarity, frequency, ambiguity and similarity with the original figure. The authors found that the adapted version was less dependent on schooling and age than the original version, and may thus be more appropriate for clinical applications. However, the test has not been standardized for populations with PD.

*Executive function tests -* The objective of the Wisconsin Card Sorting Test (WCST) is to evaluate components of the executive functions of categorizing, conceptualizing, planning, learning, perseveration of rules and successful strategies, and cognitive flexibility. This test was used in 17 studies to evaluate the main cognitive impairments in PD. The WCST has been adapted and standardized for use in Brazil and for restricted use of psychologists.^72^

Raven's Progressive Matrices (RPM) consists of a set of nonverbal tests of problem resolution involving the use of reasoning and efficacious strategies, discovery of rules and applications of mental operations. This test, used in 10 studies, has three versions: the Standard Progressive Matrices (SPM), for use with individuals of all levels of intellectual development; the Colored Progressive Matrices (CPM), for young children, elderly individuals and people with mental deficiencies; the Advanced Progressive Matrices (APM), developed for people with above average intellectual capacity, and usually indicated for university students.^59^ In Brazil, RPM has been validated only for children by Pasquali et al. (2002),^73^ who defined normative data for a population of children in the city of Porto Alegre. However, it has not yet been validated for Brazilian adults, elderly adults, or individuals with PD.

*Cognitive screening tests -* The most frequently used cognitive screening battery was the Mini Mental State Examination (MMSE), applied in 18 studies. The MMSE tests the integrity of mental functions in a rapid and simple way. It evaluates the following functions: orientation to time and place, memory, attention, calculus, language and construct ability. Scores are defined according to points, which range from 0 to 30. The variables that affect total MMSE score have been intensively discussed among researchers. Studies suggest that age and schooling of the Brazilian population have a strong influence on performance on the tasks in the MMSE. This discussion led to the validation of the MMSE^74,75^ scale for the Brazilian population and the definition of new scores according to age and schooling. At the same time, a recent study^76^ evaluated the impact of education on the MMSE subscales and items. Results revealed that schooling has no effect on naming tasks, three-stage commands, memory recall or delayed memory. Memory is a key factor in diagnosing dementia; therefore, these items may be included in the evaluation irrespective of level of education.

The Mattis dementia rating scale (MDRS), a cognitive screening battery that also evaluates general cognitive status, was used in 15 studies. This scale consists of 36 individual tasks divided into 5 subscales: attention (8 items, 37 points); initiation and perseveration (11 items, 37 points), construct (6 items, 6 points), conceptualization (6 items, 39 points) and memory (5 items, 25 points), giving a total score of 144 points. The cut-off score for absence of dementia in the Brazilian population is 122 points; scores below this level indicate a demential process.

In 2003, Porto et al.^77^ developed a Portuguese version of the MDRS and applied it to a group of individuals with Alzheimer's disease (AD), comparing them with a group of healthy elderly individuals. The authors concluded that the MDRS had good diagnostic accuracy to discriminate between patients with mild AD and control individuals. In the study population, the effects of schooling were more marked than those of age. This result was confirmed by Foss et al. (2005),^78^ who investigated the influence of low schooling and illiteracy on the evaluation of dementia by applying the MDRS. They found that schooling affects performance and concluded that illiteracy is a factor determining lower MDRS scores, which may generate diagnostic errors.

*Scales for evaluating depression symptoms -* The Beck Depression Inventory (BDI), in the form of a self-administered questionnaire, was used in 20 studies to evaluate the intensity of depression. This instrument has 21 items for symptoms and attitudes, describing behavioral, affective, cognitive and somatic signs of depression. Each item has four response alternatives in the form of statements, organized according to severity with scores that range from 0 to 3. The overall evaluation of depression is defined according to the sum of the numbers that correspond to the answers. According to the inventory, a sum of 0 to 9 is within the limits of normality, a result from 10 to 15 suggests mild depression; from 16 to 23, moderate depression, and 24 or more, severe depression.

The BDI was validated for the Brazilian population by Gorenstein and Andrade^79^ in 1986. The Hamilton Depression Rating Scale and the State-Trait Anxiety Inventory were used for comparisons: the BDI was more efficacious, and its reliability ranged from moderate to good.

Of the 89 instruments used, 20 have been tested for the Brazilian population^60,62,63,65,67,69-72,74-77,79-83,86,88,92-94^ and 12 have been standardized for populations with PD.^64,84,85,87,89-91,95^ The instruments tested for the Brazilian population and for individuals with PD are listed in Table 3.

**Table 3:** Instruments tested for the Brazilian population and for populations with PD.

Test/Scale	Instrument tested for Brazilian population	Instrument tested for population with PD
Stroop Test^63^	✓	x
Trail Making Test^64^	✓	x
Rey's Auditory Verbal Learning Test^67,69^	✓	x
Rivermead Behavioural Memory Test^82^	✓	x
Verbal Fluency^71,72^	✓	x
Boston Naming Test^73^	✓	x
Boston Diagnostic Aphasia Examination^83^	✓	x
Dex Questionnaire of the Behavioural Assessment of the Dysexecutive Syndrome^82^	✓	x
Wisconsin Card Sorting Test^72^	✓	x
Frontal Assessment Battery^83^	✓	x
Mini Mental State Examination^74-76,84^	✓	✓
Mattis Dementia Rating Scale^77,85^	✓	✓
Dementia Rating Scale^86,87^	✓	✓
Wechsler Adult Intelligence Scale^63,64^	✓	✓
Edinburgh Handedness Inventory^88^	✓	x
Beck Depression Inventory^79,89^	✓	✓
Montgomery-Asberg Depression Rating Scale^90^	x	✓
Hamilton Depression Rating Scale^90^	x	✓
State-Trait Anxiety Inventory^79^	✓	x
Hamilton Anxiety Rating Scales^91^	x	✓
Hospital Anxiety And Depression Scale^92^	✓	✓
Beck Anxiety Inventory^91^	x	✓
Bech-Rafaelsen Mania Rating Scale^93^	✓	x
Parkinson's Disease Questionnaire (PDQ-39)^94,95^	✓	✓

## DISCUSSION

The purpose of the studies included in this systematic review was to evaluate the cognition of patients with PD that underwent DBS and to investigate the impact of the neurostimulator on cognitive performance. The results showed the diversity of instruments used. There is no consensus on the use of a single test or scale, or on the cognitive functions to be evaluated.

The application of only one test is insufficient to evaluate cognition, and a group of instruments is usually applied to obtain more reliable data, given the lack of standardized instruments for PD patient groups and disagreement concerning the predominant cognitive functions in decline. According to instrument classifications, the functions most frequently evaluated were language (74.5%), memory (72.2%), attention (66.7%) and executive functions (47.7%). These functions may be impaired because of the disease, but studies in the literature draw attention to the decline of executive skills, which may be present in the initial stages of the disease.^96^ However, as demonstrated above, the use of tests for the evaluation of this function was less frequent.

Of the instruments recommended by the AAN and MDS for cognitive screening, the most frequently used were the MMSE and the MDRS. The MMSE is one of the most frequently used cognitive screening instruments for investigating cognitive decline in the elderly. However, because of the cognitive impairments in PD, studies have shown this not to be the best cognitive screening instrument for this population. Hoops et al. (2009)^84^ evaluated the discriminating validity of the MoCA and the MMSE for detecting mild cognitive impairment (MCI) and dementia in PD. They found that the MoCA had appropriate psychometric properties as a screening instrument for detecting MCI and dementia in PD, and that it is therefore more sensitive than the MMSE in this population. Along the same lines, Hanna-Pladdy et al. (2010)^97^ conducted a comparative study of the MoCA, the MMSE and the NeuroTrax battery. Results suggested that the MoCA was the most sensitive for investigating MCI in PD.

The MDRS also has good diagnostic accuracy to investigate cognitive functioning of individuals with PD. It was validated as a screening instrument for cognitive dysfunction in this population by Brown et al. (1999),^87^ and proved more sensitive to variations in the level of cognitive impairment than the MMSE. Llebaria et al. (2008)^85^ conducted a study to validate the MDRS for dementia screening in PD. Their results revealed that the MDRS has an excellent discriminating ability for diagnosing dementia in PD, and provides objective measurements.

Of the instruments proposed by the MDS for the classification of PD-D, the most often used were the DS, WCST, VF, TMT, ST, BNT, RAVLT and BDI. The recommendations made by the MDS are more extensive, including several cognitive domains for which PD patients may show decline. Since the implementation of DBS is contraindicated for PD-D, the use of various instruments to assess preoperative cognitive function is necessary for optimal indication of the procedure.

Of the main scales used to evaluate depression, mood and anxiety among individuals with PD, the BDI and the MDRS were previously evaluated to check their accuracy and correlation with clinical diagnoses by Silberman et al. (2006).^98^ The authors found positive results and suggested the use of a cut-off point of 10 for the MDRS and 18 for the BDI to help clinicians detect depression in mild and moderate PD. The use of these scales to investigate depressive symptoms was also recommended by Schrag et al. (2007),^99^ who also suggested that the BDI could be used to monitor depressive symptoms in relation to clinical or surgical treatment of PD. However, the clinical aspects of depression were not evaluated, and depression was only monitored using the BDI.

This review showed that there is no consensus on the instruments used in the evaluations of individuals with PD that underwent DBS. The AAN has recommended some instruments suitable for evaluating cognitive decline in populations with PD, but they have not been used frequently. Moreover, instruments applied should be standardized for use with this population.

The analysis of cognitive functions revealed that evaluations in this population with PD focused largely on language and memory. Studies in the literature showed that PD leads to a decline predominantly in executive functions, indicating that individuals undergoing DBS may suffer different impacts and that it is necessary to use instruments to assess both cognitive functions in order to obtain more reliable results.

Study conducted under the Post-Graduate Psychology Program - Specialization in Speech Therapy with focus on Aging (UFRGS).

## APPENDIX A

**Table:**

Data	Year (1.0)	Title	Authors/ Journal		
**Goal**	Cognitive assessment (1.0)				
**Design methodology**	Original article in humans (1.0)	Meta-analysis (1.0)	Case study (-1.0)	Literature review (-1.0)	Original article on animals and/or case study (-1.0)
**Population**	Individuals with idiopathic PD (1.0)	DBS (1.0)	Uni or bilateral (1.0)	STN or GPi (1.0)	
**Materials**	At least one cognitive test used in the study (1.0)				
**Results**	Use of resources and computer models to assess cognition (-1.0)				
**Total**	Presence of cognitive positive or negative results in the summary (1.0)		The study is expected to attain 8.0 to be included in the review		
**Repeated**	If article is identical to another in the database then it was considered void				
